# Efficacy and safety of artemether-lumefantrine and dihydroartemisinin-piperaquine for the treatment of uncomplicated *Plasmodium falciparum* malaria and prevalence of molecular markers associated with artemisinin and partner drug resistance in Uganda

**DOI:** 10.1186/s12936-021-04021-5

**Published:** 2021-12-24

**Authors:** Chris Ebong, Asadu Sserwanga, Jane Frances Namuganga, James Kapisi, Arthur Mpimbaza, Samuel Gonahasa, Victor Asua, Sam Gudoi, Ruth Kigozi, James Tibenderana, John Bosco Bwanika, Agaba Bosco, Denis Rubahika, Daniel Kyabayinze, Jimmy Opigo, Damian Rutazana, Gloria Sebikaari, Kassahun Belay, Mame Niang, Eric S. Halsey, Leah F. Moriarty, Naomi W. Lucchi, Samaly S. Svigel Souza, Sam L. Nsobya, Moses R. Kamya, Adoke Yeka

**Affiliations:** 1 Infectious Diseases Research Collaboration, Kampala, Uganda; 2 USAID’s Malaria Action Program for Districts, Kampala, Uganda; 3 National Malaria Control Division, Ministry of Health Uganda, Kampala, Uganda; 4 U.S. President’s Malaria Initiative, Kampala, Uganda; 5 Malaria Branch, Centers for Disease Control and Prevention & President’s Malaria Initiative, Atlanta, GA USA; 6 Makerere University College of Health Sciences, Kampala, Uganda

**Keywords:** Efficacy, Artemether-lumefantrine, Dihydroartemisinin-piperaquine, Malaria, Uganda

## Abstract

**Background:**

In Uganda, artemether-lumefantrine (AL) is first-line therapy and dihydroartemisinin-piperaquine (DP) second-line therapy for the treatment of uncomplicated malaria. This study evaluated the efficacy and safety of AL and DP in the management of uncomplicated falciparum malaria and measured the prevalence of molecular markers of resistance in three sentinel sites in Uganda from 2018 to 2019.

**Methods:**

This was a randomized, open-label, phase IV clinical trial. Children aged 6 months to 10 years with uncomplicated falciparum malaria were randomly assigned to treatment with AL or DP and followed for 28 and 42 days, respectively. Genotyping was used to distinguish recrudescence from new infection, and a Bayesian algorithm was used to assign each treatment failure a posterior probability of recrudescence. For monitoring resistance, *Pfk13* and *Pfmdr1* genes were Sanger sequenced and *plasmepsin-2* copy number was assessed by qPCR.

**Results:**

There were no early treatment failures. The uncorrected 28-day cumulative efficacy of AL ranged from 41.2 to 71.2% and the PCR-corrected cumulative 28-day efficacy of AL ranged from 87.2 to 94.4%. The uncorrected 28-day cumulative efficacy of DP ranged from 95.8 to 97.9% and the PCR-corrected cumulative 28-day efficacy of DP ranged from 98.9 to 100%. The uncorrected 42-day efficacy of DP ranged from 73.5 to 87.4% and the PCR-corrected 42-day efficacy of DP ranged from 92.1 to 97.5%. There were no reported serious adverse events associated with any of the regimens. No resistance-associated mutations in the *Pfk13* gene were found in the successfully sequenced samples. In the AL arm, the NFD haplotype (N86Y, Y184F, D1246Y) was the predominant *Pfmdr1* haplotype, present in 78 of 127 (61%) and 76 of 110 (69%) of the day 0 and day of failure samples, respectively. All the day 0 samples in the DP arm had one copy of the *plasmepsin-2* gene.

**Conclusions:**

DP remains highly effective and safe for the treatment of uncomplicated malaria in Uganda. Recurrent infections with AL were common. In Busia and Arua, the 95% confidence interval for PCR-corrected AL efficacy fell below 90%. Further efficacy monitoring for AL, including pharmacokinetic studies, is recommended.

*Trial registration* The trail was also registered with the ISRCTN registry with study Trial No. PACTR201811640750761

**Supplementary Information:**

The online version contains supplementary material available at 10.1186/s12936-021-04021-5.

## Background

Malaria is a major public health concern in Uganda, with over 11 million cases registered in 2019 [1]. Close to 97% of cases are caused by *Plasmodium falciparum,* and one of the fundamental strategies for malaria case management is prompt diagnosis and treatment with artemisinin-based combination therapy (ACT), as recommended by the World Health Organization (WHO) [2, 3]. In Uganda, artemether-lumefantrine (AL) is the first-line therapy and dihydroartemisinin-piperaquine (DP) the second-line therapy for uncomplicated malaria [2]. Although artesunate-amodiaquine (ASAQ) is an alternative first-line treatment, it is not currently procured by the Uganda National Malaria Control Division and its partners, although it is used widely in private medical outlets. AL and DP showed excellent treatment efficacy for uncomplicated malaria in a 2015–2016 trial in Uganda [4]. Similar observations have been made in many African countries, with PCR-corrected efficacies > 90% in most parts of the continent [5–11]. Evidence from Uganda and other countries shows that DP is associated with fewer new infections after treatment than AL, owing to piperaquine’s extended half-life [4, 11, 12].

Although ACT remains efficacious in Africa, the success of anti-malarial treatment can be affected by the presence of drug-resistant *P. falciparum* parasites, and therefore the WHO recommends periodic monitoring [13]. In ACT, the artemisinin component is short-acting and kills the majority of the parasites during the first few days of treatment, while the role of the partner drug is to eliminate the remaining parasites [14]. However, partial resistance to artemisinin derivatives, as defined by delayed parasite clearance (presence of parasitaemia in > 10% of study participants on day 3 of treatment), has been reported in recent years in some parts of Africa [14, 15] and is widespread in Southeast Asia [16]. In addition to monitoring ACT efficacy, characterization of molecular markers of resistance during a therapeutic efficacy study may complement interpretation of the clinical data. Several single nucleotide polymorphisms (SNPs) in the *P. falciparum kelch 13* (*Pfk13*) gene are associated with artemisinin partial resistance, and 10 of these SNPs have a validated association: F446I, N458Y, M476I, Y493H, R539T, I543T, P553L, R561H, P574L, and C580Y [14, 17]. Many of these mutations have been detected extensively in the Greater Mekong Sub-region [16, 18] and recently some of these, including R561H and P574L, have been detected in Rwanda (2013–2015) [15, 17] and Uganda (in 2012) [14]. In both of these studies, P574L was found in only one sample. The marker R561H was detected in 2018 in Rwanda, where 12.8% of the 218 pre-treatment samples carried R561H [15].

In the *Pfmdr-1* gene, the most commonly studied SNPs include N86Y, Y184F, S1034C, N1042D, and D1246Y. The *Pfmdr1* 86Y mutation has been associated with chloroquine and amodiaquine tolerance, while the N86 wild type codon has been implicated in decreased susceptibility to lumefantrine [19]. The 184F mutation has been associated with increased tolerance to mefloquine and artesunate [20]. The S1034C, N1042D and D1246Y mutations are associated with increased susceptibility to mefloquine, halofantrine and artemisinin derivatives [21]. A gene duplication within the *plasmepsin-2* multi-gene cluster on the parasite chromosome 14 and a non-synonymous SNP in a putative exonuclease gene (PF3D7_1362500) on chromosome 13, exo-E415G, has been associated with both in vitro piperaquine resistance and clinical treatment failures [18, 22].

Monitoring drug efficacy and resistance for early detection is required and enhanced by implementing timely treatment policies in order to mitigate this threat [14]. The focus of the present study was to assess the efficacy and safety of AL and DP while also determining the prevalence of molecular markers associated with *P. falciparum* anti-malarial resistance in the *Pfk13, Pfmdr1* and *plasmepsin-2* genes.

## Methods

### Study design

**Fig. 1:**
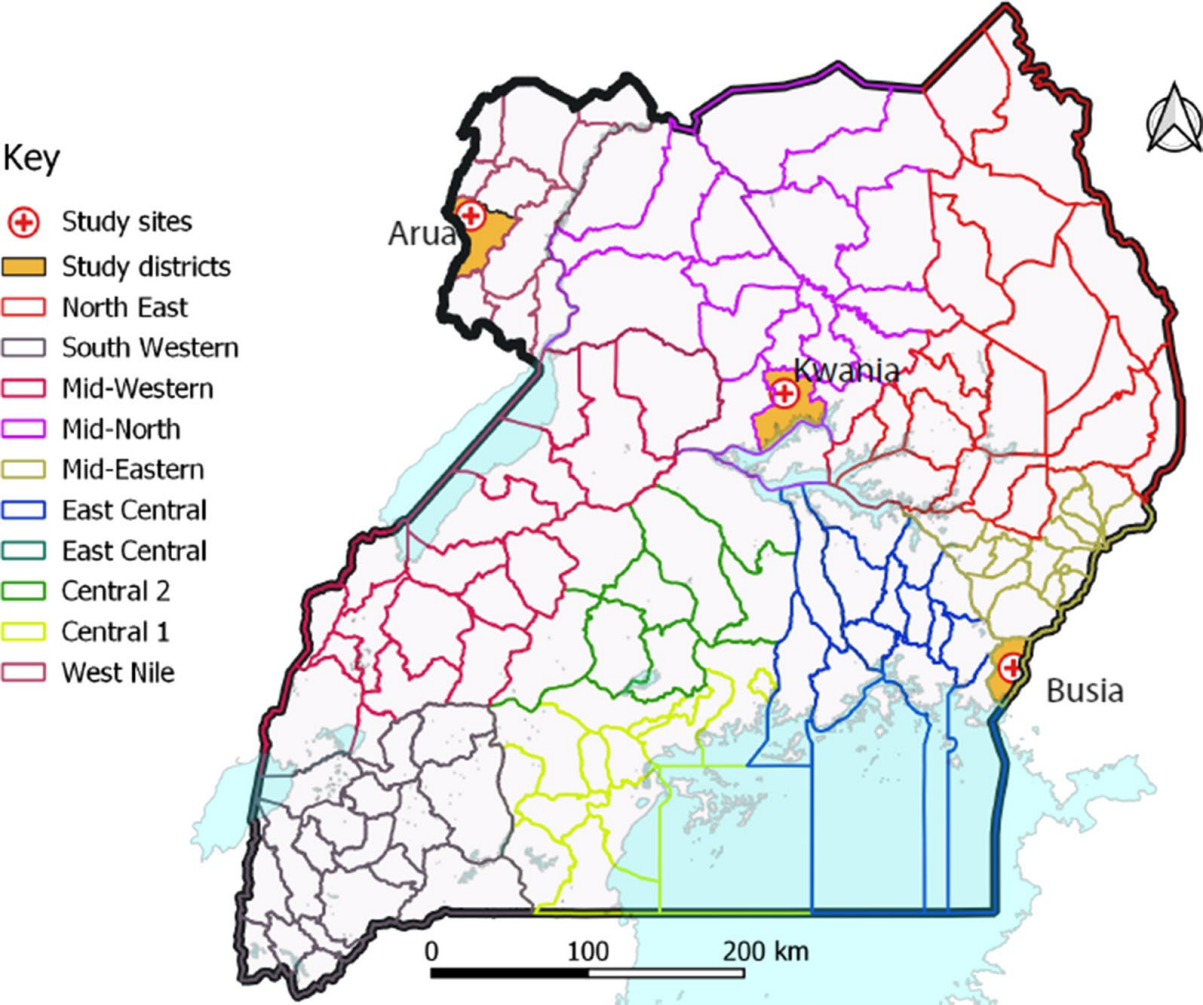
The study sites for the therapeutic efficacy study, Uganda 2018–2019

A randomized, open-label phase IV clinical trial was conducted from September 2018 to February 2019 in three health centres in Uganda: Aduku Health Centre IV in Kwania district, Northern Uganda; Arua Regional Referral Hospital in Arua district, northwestern Uganda; and, Masafu District Hospital in Busia district, Eastern Uganda (Fig. 1). All sites experience perennial malaria transmission with high transmission intensity. A 2018–2019 malaria indicator survey showed 13% parasite prevalence among children aged 0–59 months in Kwania district, 22% in Arua district and 21% in Busia district [23].

### Study population and enrolment

Inclusion criteria were children aged 6 months to 10 years with symptomatic, uncomplicated *P. falciparum* infection, with a parasite density of 2000–200,000 parasites/µl measured by microscopy; axillary temperature ≥ 37.5 °C or history of fever in the prior 24 h; weight ≥ 5 kg; absence of severe malnutrition, defined as a weight for height z-score < − 3, symmetric oedema, or mid-upper arm circumference < 110 mm; no history of serious adverse effects or hypersensitivity reactions to study medications; no regular use of medications which could interact with the study medications; no evidence of severe malaria or danger signs; no evidence of concomitant febrile illness or other known underlying chronic or severe diseases; declared consent from a parent or guardian and agreement for follow-up for 42 days; absence of repeated vomiting with the first dose of study medication; and, haemoglobin > 8.0 g/dl. Microscopic blood examination was performed by trained microscopists using thick and thin Giemsa-stained blood smears on the same slide to determine parasite density and species, respectively. Prescreening microscopy results were used for patient enrolment; definitive microscopy results were available after enrolment; patients could be excluded following enrolment based on test results.

### Randomization

A computer-generated, randomization list was created for each of the study sites by an individual not involved in the study. Sequentially numbered, sealed, opaque envelopes containing the treatment assignment were prepared and secured in a locked cabinet accessible to the study nurse. The study nurse, who was unblinded, dispensed the assigned medications but was not involved in the evaluation of participants. Patients and other study staff were informed of treatment assignments after randomization.

### Interventions

Study participants were administered one of two ACT: AL (Coartem^®^ (Novartis), Basel, Switzerland; 20 mg artemether/120 mg lumefantrine) or DP (D-Artepp^®^ (Forsun), Shanghai, China; 40 mg dihydroartemisinin/320 mg piperaquine) supplied by WHO. For both treatment arms, no food was given to the study participants, but the parents were advised to feed their children after administration of the study medication. All the doses of the study medication were administered as directly observed treatment by the study nurse at the study clinic and the study participants were monitored for 30 min after administration of study drugs. AL was administered twice a day for three days (six doses total), and DP was administered once a day for three days (three doses total). The dosage was determined using the weight-based dosage as indicated in the WHO malaria treatment guidelines [3]. If a child vomited within 30 min of administration, the medication was re-administered. Children with fever were given paracetamol and those with a haemoglobin level < 10 g/dl were treated with ferrous sulfate and anthelminthics as per Integrated Management of Childhood Illness Guidelines [24].

### Clinical follow-up

Study participants were followed daily for the first 3 days after initiation of treatment and then weekly thereafter for a total of 28 days (AL) or 42 days (DP). Study participants were also encouraged to visit the study clinic on any other day they felt unwell. At each follow-up visit, clinical response to treatment was monitored through a standardized history and physical examination, and parasitological response was assessed through examination of thick and thin blood films. Haemoglobin level was measured on day 0 and at the time of clinical treatment failure (early treatment failure (ETF) or late treatment failure (LCF). Haemoglobin was systematically measured in all patients from day 21 onwards using a portable spectrophotometer (Hemocue). A dried blood spot (DBS) was collected on the day of enrolment and on follow-up days for the parasite molecular studies.

Treatment failures, as defined by WHO [13], were treated with quinine tablets (10 mg/kg) every eight hours for five days. Patients with evidence of severe malaria or danger signs (including haemoglobin < 5 g/dl, convulsions, lethargy, inability to drink or breastfeed, repeated vomiting, and inability to sit/stand because of weakness) were referred for treatment with parenteral artesunate. Participants who took anti-malarial medications outside the study, experienced adverse events requiring a change in treatment, withdrew informed consent, or were lost to follow-up were excluded at the time of these events.

### Malaria microscopy

Malaria parasitaemia was diagnosed using thick smears stained for 10 min with 10% Giemsa. Follow-up thick and thin smears were stained with 2% Giemsa for 30 min and used to determine the actual parasite density, species and presence of gametocytes. Parasitaemia was measured by counting the number of asexual parasites against 200 leucocytes in thick blood films, and thin films were used for detection of the different parasite species. Parasite density per µl of blood was calculated by multiplying the total number of parasites counted by 40, assuming that 1 µl of blood had a mean count of 8000 leucocytes [25, 26]. When more than 500 parasites were identified before counting 200 leucocytes, counting was stopped and parasitaemia was calculated using the actual number of leucocytes counted. A blood smear was declared negative when examination of 100 high power fields did not reveal the presence of malaria parasites. For quality control, each smear was read by two microscopists, with disagreements defined as differences between the two microscopists in species diagnosis, in parasite density of > 50%, or in the presence of parasites, settled by a third microscopist. The final parasitaemia was calculated as the average between the two readings for readings without disagreement. For readings with disagreement, the final parasitaemia was calculated as the average of the third microscopist’s and the closest of the first two readings; the third microscopist’s reading was taken as the final species.

### Molecular analysis

Molecular markers of anti-malarial drug resistance and microsatellite markers were analysed at the Centers for Disease Control and Prevention (CDC) Malaria Laboratory in Atlanta, USA [27, 28]. Parasite genomic DNA was extracted from DBS collected on day 0 and day of recurrent infection using the QIAamp blood mini-kits (Qiagen GmbH, Hilden, Germany) according to the manufacturer’s instructions [29]. The PET-PCR assay [30] was used to analyse the quality of the extracted parasite genomic DNA and to confirm the presence of *P. falciparum*.

Paired samples with recurrent infections (day 0 and day of recurrent infection) were analysed to distinguish recrudescence from re-infection using seven neutral microsatellite markers (TA1, Poly-α, PfPK2, TA109, TA2490, C2M34, C3M69) over six chromosomes [31, 32]. Fragment size was measured by capillary electrophoresis on ABI 3033 (Applied Biosystems) and scored using GeneMarker^®^ V2.6.3 (SoftGenetic, LLC, PA, USA). A Bayesian probabilistic algorithm was used to distinguish recrudescent from new infections, accounting for classification uncertainty with multi-parasite genetic diversity [28, 33].

Sanger sequencing was used to investigate *Pfk13* propeller domain (codon positions: 389-649), as previously described [34], in all the day 0 and day of recurrent infection samples in both study arms. The *Pfmdr1* (codon positions: 86, 184, 1034, 1042, 1246) mutations were investigated in paired day 0 and day of recurrent infection samples in the AL arm using a previously described method [35]. SNPs were identified using the Geneious software package (Biomatters, Inc., San Francisco, CA, USA). The 3D7 *Pfk13* and *Pfmdr1* were used as reference sequences. Heterozygous SNPs (double peaks representing mixed infections) were identified using the heterozygous caller plug-in tool in Geneious with a minor allele threshold of at least 30%. For samples with mixed infections and SNP variations at multiple sites, each possible haplotype constructed from the observed SNPs was reported for *Pfmdr1*. Detection of *P. falciparum plasmepsin 2* copy number was performed in paired day 0 and recurrent infection samples in the DP arm using an Agilent Mx3005 real-time PCR machine (Agilent Technologies, CA, USA), according to previously described protocols [36]

### Outcomes

Parasitaemia, assessed by microscopy within 28 (AL and DP) or 42 (DP) days of treatment, was either unadjusted or PCR-adjusted to distinguish recrudescence from new infection, and was used to calculate outcomes. Recrudescence was defined as the recurrence of asexual parasitaemia of the same genotype(s) that caused the original illness, due to incomplete clearance of asexual parasites after anti-malarial treatment, while new infection was an infection that followed a primary infection which is often (but not always) different from that which caused the initial infection [13, 14].

Outcomes were classified according to WHO guidelines [13] and included: ETF, danger signs, complicated malaria, or failure to adequately respond to therapy on days 0–3; LCF: danger signs, complicated malaria, or fever and parasitaemia on days 4-28/42); late parasitological failure (LPF): asymptomatic parasitaemia on days 7-28/42; and, adequate clinical and parasitological response (absence of parasitaemia through follow-up). Secondary outcomes included prevalence of fever and parasitaemia on days 1–3, the prevalence of gametocytes during follow-up, risk of adverse events, and presence of genetic polymorphisms associated with anti-malarial drug resistance. Adverse events were evaluated at each study visit, graded according to WHO and US National Institute of Allergy and Infectious Diseases scales, and defined based on International Conference on Harmonization guidelines as untoward medical occurrences. Serious adverse events included death, a life-threatening experience, hospitalization, incapacity, or events that required medical or surgical intervention to prevent serious outcomes [37, 38].

### Sample size

Both AL and DP were studied at each site, with a target of 100 study participants in each treatment arm. The sample size calculation was based on estimated risks of treatment failure (outcomes classified as ETF, LCF or LPF) of the treatment regimens to be studied [13]. Based on prior data, the risk of treatment failure for both AL and DP were estimated at 5% with a 5% margin of error and 95% confidence interval. The calculated sample size was adjusted by 35% to account for recurrent infection rates, thus the total target sample size per site per treatment arm was 100.

### Data management and statistical methods

Data were double-entered into Microsoft Access and analysed using Stata, version 14.2 (Stata). Per-protocol (proportional) and cumulative efficacy were calculated by arm and site at 28- or 42-days follow-up. Unadjusted outcomes were censored for loss to follow-up or exclusion. Outcomes adjusted by genotyping were censored for loss to follow-up or exclusion, new infections or failure of genotyping. The posterior probabilities of recrudescence generated using the Bayesian algorithm to distinguish recrudescence from re-infection were used to generate the per-protocol (proportional) efficacies, and posterior sampling was used to generate the Kaplan–Meier estimates and 95% confidence intervals. For Markers *Pfmdr1* gene and *plasmepsin-2* gene copy number, samples from subjects who failed therapy were evaluated, and for *Pfk13,* samples from all subjects were evaluated. Incident adverse events were reported by arm.

## Results

### Trial profile

**Fig. 2:**
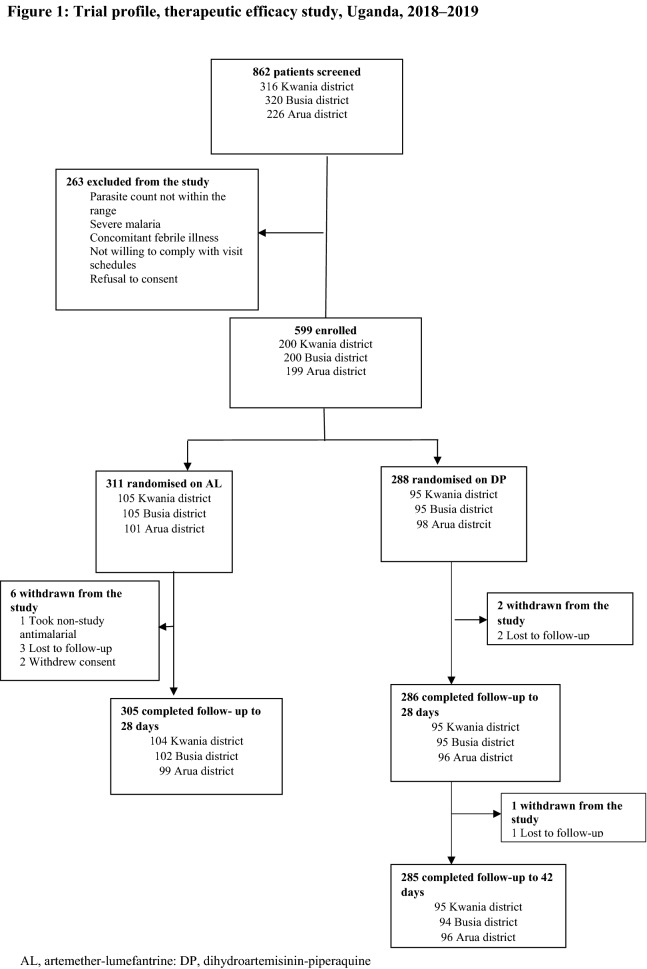
Trial profile, therapeutic efficacy study, Uganda, 2018–2019

Among 862 patients screened between September 2018 and February 2019, 263 were excluded during screening and 599 were randomized to receive therapy with either AL or DP; baseline characteristics are shown in Table 1. Nine patients were withdrawn from the study during the follow-up period. An outcome was reached for 590 study participants, representing 98.5% of the total enrolled study participants (Fig. 2).

**Table 1:** The baseline characteristics of the study participants enrolled during the therapeutic efficacy study in Uganda 2018–2019

Characteristics, location	Treatment arm	
	AL (N = 305)	DP (N = 285)
Female sex n/N (%)		
Kwania district	59/104 (56.7)	54/95 (56.8)
Busia district	63/102 (61.8)	48/94 (51.1)
Arua district	48/99 (48.5)	56/96 (58.3)
Age in years, median (range)		
Kwania district	6.0 (1.0–10.0)	6.0 (1.0–10.0)
Busia district	2.0 (0.5–10.0)	3.0 (0.5–10.0)
Arua district	3.0 (0.5–10.0)	3.0 (0.8–10.0)
Temperature, °C, mean (SD)		
Kwania district	37.4 (1.3)	37.4 (1.2)
Busia district	38.0 (0.9)	37.8 (0.9)
Arua district	38.4 (1.0)	38.1 (1.0)
Parasite density/μl, Geometric mean, (95% CI)		
Kwania district	36,910 (29,193–46,666)	30,906 (24,067–39,687)
Busia district	35,073 (28,235–43,567)	29,142 (22,643–37,506)
Arua district	43,536 (34,973–54,194)	45,083 (35,329–57,530)
Hemoglobin level, g/dl, mean (SD)		
Kwania district	11.4 (1.4)	11.4 (1.5)
Busia district	11.5 (1.7)	11.7 (1.9)
Arua district	11.1 (1.7)	11.4 (1.6)

AL, artemether-lumefantrine; DP, dihydroartemisinin-piperaquine; SD, standard deviation; CI, confidence interval

### Efficacy of the two anti-malarial regimens

**Fig. 3:**
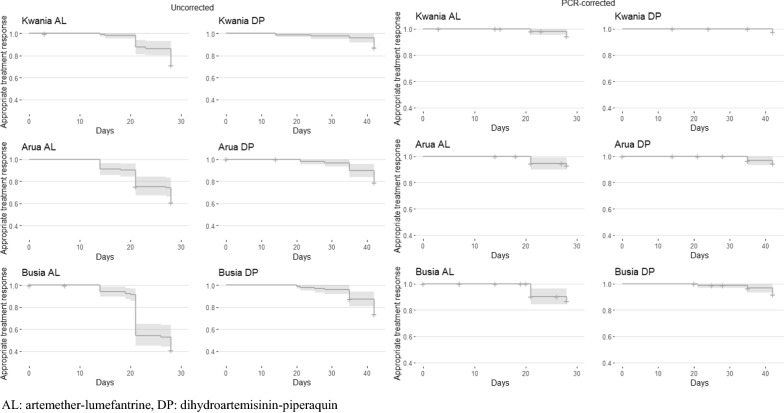
Uncorrected and PCR-corrected Kaplan–Meier survival curves of time to treatment failure among participants enrolled in therapeutic efficacy monitoring, Uganda, 2018–2019. *AL* artemether-lumefantrine, *DP* dihydroartemisinin-piperaquin

The primary efficacy outcomes are shown in Table 2. There were no ETFs. PCR-correction was conclusive for 185 out of 186 samples that were eligible (99.5%). The uncorrected 28-day cumulative efficacy of AL ranged from 41.2 to 71.2% and the PCR-corrected cumulative 28-day efficacy of AL ranged from 87.2 to 94.4%. The uncorrected 28-day cumulative efficacy of DP ranged from 95.8 to 97.9% and the PCR-corrected cumulative 28-day efficacy of DP ranged from 98.9 to 100%. The uncorrected 42-day efficacy of DP ranged from 73.5 to 87.4% and the PCR-corrected 42-day efficacy of DP ranged from 92.1 to 97.5%. Kaplan–Meier survival estimates are depicted in Fig. 3 and raw genotyping data are available in Additional file 1: Table S1.

**Table 2:** Primary efficacy outcomes, Uganda therapeutic efficacy monitoring, 2018–2019

Outcome	Kwania district			Arua district			Busia district		
	AL (28 days)	DP (28 days)	DP (42 days)	AL (28 days)	DP (28 days)	DP (42 days)	AL (28 days)	DP (28 days)	DP (42 days)
ETF n (%)	0 (0.0)	0 (0,0)	0 (0.0)	0 (0.0)	0 (0.0)	0 (0.0)	0 (0.0)	0 (0.0)	0 (0.0)
LCF n (%)	5 (4.8)	1 (1.0)	2 (2.1)	11 (11.1)	0 (0.0)	0 (0.0)	13 (12.7)	2 (2.1)	4 (4.2)
LPF n (%)	25 (24.0)	1 (1.0)	10 (10.5)	28 (28.3)	3 (3.1)	20 (20.8)	47 (46.1)	2 (2.1)	21 (22.3)
ACPR n (%)	74 (71.2)	93 (97.9)	83 (87.4)	60 (60.6)	93 (96.9)	76 (79.2)	42 (41.2)	91 (95.8)	69 (73.4)
Reinfection^a^ n (%)	25 (83.3)	2 (100)	10 (83.3)	32 (82.1)	3 (100)	15 (75.0)	50 (83.3)	3 (75.0)	18 (72.0)
Recrudescence^b^ n (%)	5 (16.7)	0 (0.0)	2 (16.7)	7 (17.9)	0 (0.0)	5 (25.0)	10 (16.7)	1 (25.0)	7 (28.0)
Uncorrected Kaplan Meier % [95% CI]	71.2 [63, 80]	97.9 [95, 100]	87.4 [81, 94]	60.6 [52, 71]	96.9 [94, 100]	79.2 [71, 88]	41.2 [33, 52]	95.8 [92, 100]	73.5 [65, 83]
Corrected Kaplan Meier % [95% CI]	94.4 [90, 99]	99.5 [99, 100]	97.5 [95, 100]	92.1 [86, 98]	100.0 [100, 100]	94.4 [90, 99]	87.2 [79, 95]	98.9 [97, 100]	92.1 [87, 98]
Uncorrected per protocol efficacy % [95% CI]	71.2 [61, 80]	97.9 [91, 100]	87.4 [79, 93]	60.6 [50, 70]	96.9 [88, 98]	79.2 [70, 87]	41.2 [32, 51]	95.8 [90, 99]	73.4 [63, 82]
Corrected per protocol efficacy % [95% CI]	93.1 [85, 98]	99.5 [95, 100]	97.3 [91, 100]	89.7 [80, 96]	100.0 [96, 100]	93.7 [86, 98]	80.1 [67, 90]	98.9 [94, 100]	90.8 [82, 96]

ETF, early treatment failure; LCF, late clinical failure; LPF, late parasitological failure, ACPR, adequate clinical and parasitological response; AL, artemether-lumefantrine; DP, dihydroartemisinin-piperaquine

^a^For tabulation, reinfection defined as a recurrent infection with a posterior probability of recrudescence of < 50%

^b^For tabulation, recrudescence defined as a recurrent infection with a posterior probability of recrudescence of ≥ 50%

Secondary outcomes included the proportion of patients with parasites on days 1–3 and proportion of patients with fever after therapy (Table 3). Parasitaemia on day 3 was uncommon for DP, with one (1.1%) in Kwania district, and two (2.1%) in Arua district, and AL, with three (3.0%) in Arua district. The proportion of patients with fever on day 3 was low for both DP, with one (1.1%) in Kwania district, and AL, with two (2.0%) in Busia district.

**Table 3:** Secondary efficacy outcomes, Uganda therapeutic efficacy monitoring 2018–2019

Secondary Efficacy outcome	Kwania district		Arua district		Busia district	
	AL	DP	AL	DP	AL	DP
Parasitemia prevalence n (%)						
Day 1	72 (69.2)	65 (68.4)	98 (99.0)	88 (91.7)	93 (91.2)	75 (79.8)
Day 2	14 (13.5)	11 (11.6)	46 (46.5)	31 (32.3)	9 (8.8)	7 (7.5)
Day 3	0 (0.0)	1 (1.1)	3 (3.0)	2 (2.1)	0 (0.0)	0 (0.0)
Fever prevalence n (%)						
Day 1	2 (1.96)	4 (4.2)	6 (6.13)	9 (9.3)	5 (4.9)	2 (2.1)
Day 2	0 (0.0)	1 (1.1)	0 (0.0)	1 (1.0)	4 (3.9)	0 (0.0)
Day 3	0 (0.0)	1 (1.1)	0 (0.0)	0 (0.0)	2 (2.0)	0 (0.0)
Gametocyte prevalence n (%)						
Day 1	1 (1.0)	1 (1.1)	4 (4.0)	6 (6.3)	11 (10.8)	15 (16.0)
Day 2	0 (0.0)	1 (1.1)	4 (4.0)	6 (6.3)	5 (4.9)	13 (13.8)
Day 3	0 (0.0)	0 (0.0)	3 (3.0)	6 (6.3)	3 (2.9)	12 (12.8)
Day 4–28 (AL) or 42 (DP)	0 (0.0)	0 (0.0)	0 (0.0)	1 (1.0)	0 (0.0)	1 (0.1)

AL, artemether-lumefantrine; DP, dihydroartemisinin-piperaquine

### Adverse events

The most common adverse events were cough, acute watery diarrhoea, and mild skin rash (Table 4). Two serious adverse events, fracture on day 11 and severe malaria on day 23, occurred among study participants in the AL treatment arm. The severe malaria case was treated using artesunate based on Uganda guidelines for the management of severe malaria. All adverse events resolved without any sequelae during the study period. None of the adverse events was judged to relate to the study medications.

**Table 4:** Frequency of adverse events reported during the therapeutic efficacy monitoring in Uganda at all sites, 2018–2019

	Artemether-lumefantrine, n (%)	Dihydroartemisinin-piperaquine, n (%)
Adverse events		
Cough	119 (39.0)	122 (42.8)
Acute watery diarrhea	43 (14.1)	33 (11.6)
Mild skin rash	15 (4.9)	14 (4.9)
Mild anemia (8.0–10.0 g/dl)	16 (5.2)	15 (5.3)
Serious adverse events		
Fracture	1 (0.3)	0 (0.0)
Severe malaria	1 (0.3)	0 (0.0)

### Prevalence of *Plasmodium falciparum* genetic polymorphisms mediating anti-malarial resistance

*Plasmodium falciparum* genetic polymorphisms associated with anti-malarial susceptibility in parasites isolated at enrolment (day 0) and at the time of recurrent infection were assessed. No artemisinin partial resistance-associated mutations in the *Pfk13* gene was found in the 338/370 (91.4%) successfully sequenced paired day 0 and day of recurrent infections samples. The *Pfmdr1* gene was investigated in paired day 0 and day of recurrent infection samples in the AL study arm. SNP results for each of the investigated codons are reported in Table 4. None of the successfully sequenced samples had mutations in codons 86, 1034 and 1042. The *Pfmdr1* haplotype construction was done using data for codons 86, 184 and 1246 in samples that had complete data at these codons (Table 5). NFD and NYD haplotypes, associated with tolerance to lumefantrine, were observed in 65 and 62% of the samples, respectively (Table 5). A total of 29 samples in the DP arm, 19 from day 0 and 10 from day of recurrent infection, had a multiplicity of infection (MOI) of one and were successfully investigated for *plasmepsin-2* gene copy number variation, and all the 29 had one copy of the *plasmepsin-2* gene.

**Table 5:** Prevalence of *Pfmdr1* alleles and haplotypes in day 0 (pre-treatment) and day of recurrent infection samples in the artemether-lumefantrine arm, 2018–2019 therapeutic efficacy monitoring

^a^*Pfmdr1* codon	Day 0	Day of recurrent infection
N86	128 (100%)	127 (100%)
86N/Y (N = 255)	0 (0%)	0 (0%)
86Y	0 (0%)	0 (0%)
Y184	48 (38%)	38 (32%)
Y184Y/F (N = 246)	37 (29%)	38 (32%)
184F	43 (34%)	42 (36%)
S1034	126 (100%)	111 (100%)
1034S/C (N = 237)	0 (0%)	0 (0%)
1034C	0 (0%)	0 (0%)
N1042	127 (100%)	111 (100%)
1042N/D (N = 238)	0 (0%)	0 (0%)
104D	0 (0%)	0 (0%)
D1246	116 (91%)	107(96%)
1246D/Y (N = 239)	5 (4%)	2(2%)
1246Y	6 (5%)	3 (3%)
*Pfmdr1* haplotypes^b^	n = 127^c^	n = 110^c^
NFD	78 (61%)	76 (69%)
NYD	79 (62%)	68 (62%)
YFD	0	0
YYD	0	0
YYY	0	0
NYY	9 (7%)	4 (4%)
YFY	0	0
NFY	6 (5%)	3 (3%)

^a^Data presented are for participants with either later clinical failure or late parasitological failure

^b^Each possible haplotype constructed from the mixed infections (wildtype and mutant) was reported. Haplotype percentages exceed a sum of 100% because all possible haplotypes from mixed infections (both wild type and mutants) were included in the construction of haplotypes

^c^N includes samples for which data were available for all three markers

## Discussion

This study was conducted as a routine therapeutic efficacy and safety monitoring of AL and DP for the treatment of uncomplicated malaria in Uganda at three sites. The unadjusted efficacy for AL was low, with Busia district having approximately half of the children returning to the clinic with parasitaemia within 28 days. The PCR-corrected efficacy of AL was below the 90% WHO threshold or the 95% confidence interval crossed the 90% threshold in two of three sites. DP offered acceptable treatment efficacy and had fewer than 30% recurrent infections in all the sites.

In this study, no *Pfk13* mutations associated with artemisinin resistance were detected, although only codons between 389 and 649 was examined. Data from other studies in Uganda on *Pfk13* propeller domain mutations associated with artemisinin delayed clearance showed that the prevalence of 469Y and 675 V mutations has increased at multiple sites in northern Uganda (up to 23 and 40%, respectively) [39]. There is need for continued surveillance of *Pfk13* polymorphisms and their possible association with reduced efficacy of artemisinin in Uganda. Analysis of the *Pfmdr1* gene showed that NFD (N86/184F/D1246) and NYD (N86/Y184/D1246), both associated with reduced susceptibility to lumefantrine, were the most common haplotypes observed.

Prior studies in Uganda have consistently shown high efficacy for DP and AL [4, 40]. The low efficacy of AL in Busia district is an early warning of a possible onset of resistance to ACT in Uganda after over a decade of use. There has been reported emergence of de novo parasite genetic markers for artemisinin partial resistance in Rwanda after the use of AL for approximately a decade [15]. Despite the low cumulative efficacy for AL noted in Busia district, the parasite prevalence on day 3 was far below the 10% WHO threshold for the definition of suspected partial artemisinin resistance [41]. These results suggest that clinically relevant resistance to artemisinin continues to be absent in Uganda, consistent with recent reports based on ex vivo drug susceptibility [42, 43]. The increasing prevalence of wild-type isolates with *Pfmdr1* N86 polymorphisms associated with decreased sensitivity to lumefantrine could be contributing to the high levels of recurrent parasitaemia in the AL treatment arm.

As reported in prior studies, treatment with DP provides a longer period of post-treatment prophylaxis than AL [44], leading to a lower incidence of recurrent malaria within 28 days of therapy. This, coupled with a once-daily dosing scheme and no requirement to administer with a fatty food, makes DP an attractive option for the treatment of malaria in high-transmission settings such as Uganda [23]. With the 95% confidence interval for PCR-corrected efficacy of AL falling below the 90% WHO threshold in some sites, there is potential for DP to be considered as first-line treatment of malaria in Uganda. The efficacy of the newest anti-malarial regimen included in the national policy, pyronaridine-artesunate, should be monitored as well to provide efficacy data on all treatment options in the country.

This is the first time that seven neutral microsatellites were used for molecular correction of therapeutic efficacy monitoring results in Uganda, which may partially account for the variation in efficacy between this study and past studies. Previous studies in Uganda used *msp1, msp2* and up to four microsatellites to differentiate recrudescent from new infections [4, 40]. The Bayesian algorithm for microsatellite interpretation was validated and found to have high specificity and generated accurate failure rate estimates [33]. The Bayesian method used in this study is particularly useful in a highly diverse parasite population with an increased MOI because some data may be missing during molecular correction and the Bayesian methods account for this uncertainty [33]. With this findings, further routine efficacy monitoring with increased sample size for the AL treatment arms to account for the high re-infection rate is recommended.

One limitation of this study was that co-administration of AL with a fatty food, as recommended by the product insert, was not consistently enforced. Future studies should ensure a fatty food is provided when administering AL and incorporate laboratory analysis of lumefantrine level to determine whether low drug levels are contributing to low efficacy findings. Another limitation was the expanded age range, 6 months to 10 years, due to low transmission in some study areas. The confounding factor of acquired immunity, which would be more probable in older children, may have resulted in overestimation of efficacy compared with studies recruiting children 6–59 months.

## Conclusions

DP offered PCR-corrected efficacy greater than 90% in all three study sites. The point estimate for the PCR-corrected efficacy for AL was below 90% in some areas. There were no *Pfk13* mutations or increased *plasmepsin-2* copy number associated with artemisinin or piperaquine resistance, respectively. Although both AL and DP are still considered appropriate for the treatment of uncomplicated malaria by the Uganda National Malaria Control Division, there is continued need for routine monitoring of the efficacy of anti-malarials, particularly for AL in Busia district.

## Supplementary Information

**Additional file 1: Table S1.** Raw genotyping data.

## Data Availability

The datasets used during the current study are available from the corresponding author upon request.

## Abbreviations

ABI: Applied biosystems
ACPR: Adequate clinical and parasitological response
ACT: Artemisinin-based combination therapy
AL: Artemether-lumefantrine
ASAQ: Artesunate-amodiaquine
CDC: Centers for Disease Control and Prevention
DBS: Dried blood spot
DP: Dihydroartemisinin-piperaquine
ETF: Early treatment failure
LCF: Late treatment failure
LPF: Late parasitological failure
MOI: Multiplicity of infection
PCR: Polymerase chain reaction
PET-PCR: Photo-induced electron transfer polymerase chain reaction
Pfk13: Plasmodium falciparum Kelch 13
Pfmdr1: Plasmodium falciparum multidrug resistance 1 transporter
qPCR: Quantitative polymerase chain reaction
SNP: Single nucleotide polymorphisms
WHO: World Health Organization
