# Correction to: Efficacy and safety of artemether-lumefantrine and dihydroartemisinin-piperaquine for the treatment of uncomplicated *Plasmodium falciparum* malaria and prevalence of molecular markers associated with artemisinin and partner drug resistance in Uganda

**DOI:** 10.1186/s12936-022-04049-1

**Published:** 2022-02-07

**Authors:** Chris Ebong, Asadu Sserwanga, Jane Frances Namuganga, James Kapisi, Arthur Mpimbaza, Samuel Gonahasa, Victor Asua, Sam Gudoi, Ruth Kigozi, James Tibenderana, John Bosco Bwanika, Agaba Bosco, Denis Rubahika, Daniel Kyabayinze, Jimmy Opigo, Damian Rutazana, Gloria Sebikaari, Kassahun Belay, Mame Niang, Eric S. Halsey, Leah F. Moriarty, Naomi W. Lucchi, Samaly S. Svigel Souza, Sam L. Nsobya, Moses R. Kamya, Adoke Yeka

**Affiliations:** 1 Infectious Diseases Research Collaboration, Kampala, Uganda; 2 USAID’s Malaria Action Program for Districts, Kampala, Uganda; 3 National Malaria Control Division, Ministry of Health Uganda, Kampala, Uganda; 4 U.S. President’s Malaria Initiative, Kampala, Uganda; 5 Malaria Branch, Centers for Disease Control and Prevention & President’s Malaria Initiative, Atlanta, GA USA; 6 Makerere University College of Health Sciences, Kampala, Uganda

## Correction to: Malaria Journal (2021) 20:484 https://doi.org/10.1186/s12936-021-04021-5

Following publication of the original article [1], it was brought to the publisher’s attention that incorrect details had been provided in the trial registration information of the article.

Where it should read:

- Trial registration: The trial was also registered with the Pan African Clinical Trial Registry (https://pactr.samrc.ac.za/) with study Trial No. PACTR201811640750761.

It said:

- Trial registration: The trial was also registered with the ISRCTN registry with study Trial No. PACTR201811640750761.

The original article has been updated accordingly.
